# Skewness of X-chromosome inactivation increases with age and varies across birth cohorts in elderly Danish women

**DOI:** 10.1038/s41598-021-83702-2

**Published:** 2021-02-22

**Authors:** Jonas Mengel-From, Rune Lindahl-Jacobsen, Marianne Nygaard, Mette Soerensen, Karen Helene Ørstavik, Jens Michael Hertz, Karen Andersen-Ranberg, Qihua Tan, Kaare Christensen

**Affiliations:** 1 Epidemiology, Biostatistics and Biodemography, the Danish Twin Registry, and the Danish Aging Research Center, Department of Public Health, University of Southern Denmark, J.B. Winsløws Vej 9, 5000 Odense C, Denmark; 2 Department of Clinical Genetics, Odense University Hospital, Odense, Denmark; 3 Department of Clinical Biochemistry and Pharmacology, Odense University Hospital, Odense, Denmark; 4 Institute of Clinical Medicine, University of Oslo, Oslo, Norway; 5 Geriatric Research Unit, Department of Clinical Research, University of Southern Denmark, Odense, Denmark; 6 Interdisciplinary Centre on Population Dynamics (CPop), University of Southern Denmark, Odense, Denmark

**Keywords:** Epigenetics, Genetic markers, Genomic instability, Molecular medicine

## Abstract

Mosaicism in blood varies with age, and cross-sectional studies indicate that for women, skewness of X-chromosomal mosaicism increases with age. This pattern could, however, also be due to less X-inactivation in more recent birth cohorts. Skewed X-chromosome inactivation was here measured longitudinally by the HUMARA assay in 67 septuagenarian and octogenarian women assessed at 2 time points, 10 years apart, and in 10 centenarian women assessed at 2 time points, 2–7 years apart. Skewed X-chromosome inactivation was also compared in 293 age-matched septuagenarian twins born in 1917–1923 and 1931–1937, and 212 centenarians born in 1895, 1905 and 1915. The longitudinal study of septuagenarians and octogenarians revealed that 16% (95% CI 7–29%) of the women developed skewed X-inactivation over a 10-year period. In the cross-sectional across-birth cohort study, the earlier-born septuagenarian (1917–1923) and centenarian women (1895) had a higher degree of skewness than the respective recent age-matched birth cohorts, which indicates that the women in the more recent cohorts, after the age of 70, had not only changed degree of skewness with age, they had also undergone less age-related hematopoietic sub-clone expansion. This may be a result of improved living conditions and better medical treatment in the more recent birth cohorts.

## Introduction

Mosaicisms in peripheral blood occur in various forms and may or may not vary with age. Some common mosaicisms are known to increase with age, e.g. loss of sex chromosomes (Y or X)^1,2^, clonal hematopoiesis of indeterminate potential^3,4^, and skewness of X-inactivation^5^. However, these studies are mainly based on cross-sectional studies and, hence, the observed patterns could be confounded by birth cohort differences. For example, if more recent birth cohorts experienced less mosaicism compared to previous birth cohorts because of better environmental conditions, less infection load, better lifestyle and/or better available health care, such cohort differences could occur.

A clear birth cohort effect is observed for cognitive abilities in Danes born in 1915. This cohort performed cognitively better as nonagenarians than did the 1905 cohort a decade earlier, illustrating the Flynn effect at high ages^6^. In contrast, hand grip strength is shown to be similar in the two cohorts^6^; however, it varies greatly between men and women and declines with age for both genders in cross-sectional as well as longitudinal studies^7^. Longevity gene variants also vary by frequency between oldest-old cohorts, with a moderate decrease in allele frequencies of *APOE* and *FOXO3A* variants in individuals from more recent birth cohorts^8^.

Across a variety of mammalian species, X-chromosome inactivation exists as a sex chromosome dosage compensation that occurs between the sexes on the X-chromosome^9^. Basically, in females, one of the two X-chromosomes, either the maternal or the paternal, is inactivated in early embryonic life at about the time of implantation^10^. This ensures that the basal gene expression in each cell is equally dosed in women and men. In cell populations in females, such as a pool of leukocytes, two lines are therefore defined by either the maternal X-chromosome or the paternal X-chromosome being the inactive X-chromosome. Most women are said to be mosaics as the 2 cell lines are commonly distributed nearing a 50:50 ratio^11^. Divergence from this equal balance of the 2 cell lines in younger women is related to genetic disorders, e.g. skewed X inactivation may explain the manifestation of X-linked disorders in women. Traditionally the HUMARA assay is used for measures of X-chromosome inactivation, also termed as degree of skewness (DS)^12^. For patients and their family members with rare X-linked disease alleles, DS in women indicates whether one of the two alleles is more abundant than the other, and the mosaicism state is thus skewed, usually with an 80:20 ratio or more^13^. DS increases considerably with age in cross-sectional studies of populations older than 55–60 years, and this age association continues to increase at the most advanced ages^5,11,14–18^. Although evidence firmly establishes that skewed X-inactivation increases with age cross-sectionally, it is less certain whether skewed X-inactivation increases with age longitudinally, as only few studies have investigated X-inactivation repeatedly in the same sample of women over time. The X-inactivation pattern analyzed in peripheral granulocytes, monocytes and T-cells in 35 blood donors aged 20–75 years was reported not to be significantly different in samples drawn 18 months apart^19^. The authors thus stated that no fluctuation of X-inactivation pattern occurred in these blood cells during this time. Similarly, no differences were found between samples from 178 females aged 5 to 75 years drawn two decades apart. Only in the few samples where the age at the time of the second sampling was more than 60 years, a tendency towards a more skewed X-inactivation in the second sample was found^15^. In a sample of 45 pairs of twin girls, a minor increase in the prevalence of moderate skewing (> 30%) was observed from age 5 to 10^20^. Although skewed X-inactivation may start at a young age, the skewing was considerably lower than what is normally considered skewed X-inactivation in clinical genetics^5^.

Skewed X-inactivation is most often measured in blood, but the heritability of skewed X-inactivation in female twins is dependent on the tissue, and while skewed X-inactivation increases with age in blood, it appears fixed with age in fat and skin tissue^18^. We previously demonstrated that skewed X-inactivation was associated with mortality, and, unexpectedly, women who had higher DS survived longer than women with normal mosaicisms^21^. The hypothesis of selection by mortality may partly explain the increase in skewed X-inactivation with temporal age, but also cohort effects may play a role in age-related differences. Older cohorts have a higher selection pressure throughout life^22^ and younger cohorts may be biologically younger than older cohorts^23^. Factors that might drive skewed X-inactivation include DNA modifications. Recent advances in sequencing technology have revealed an age-related increase in clonal hematopoiesis that might be related to skewed X-inactivation; e.g., somatic mutations in the *TET2* gene are observed among elderly women with skewed X-inactivation^24,25^.

In this study we tested, using repeated measures, whether skewed X-inactivation changes with age in whole blood from elderly twins and singleton centenarians, and whether skewed X-inactivation patterns vary between birth cohorts of elderly women.

## Results

### Increase in skewed X-inactivation with age

**Figure 1:**
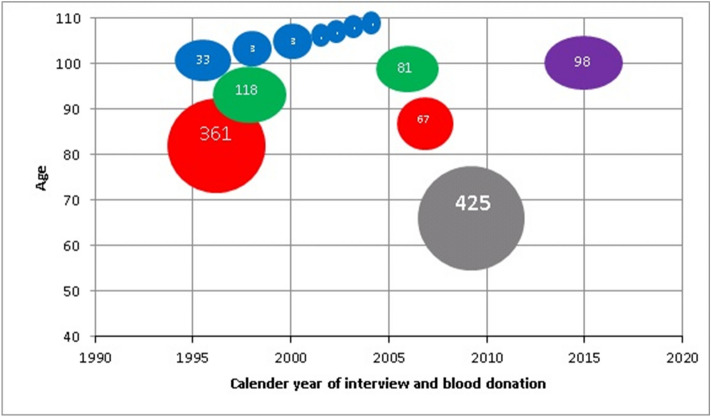
Lexis diagram of populations of women with measured degree of skewed X-inactivation. Blue: 1895 birth cohort with repeated measures at the latest follow-up participation. Green: 1905 birth cohort (cross-sectional). *Note* There are no repeated measures, thus the 1905 cohort is not included in longitudinal studies. Purple: 1915 birth cohort (cross-sectional). Red: LSADT with repeated measures at follow-up. Gray: MADT (cross-sectional).

A study population of septuagenarian and octogenarian female twins donated blood both at baseline and after 10 years of follow-up as displayed in Table 1 and Fig. 1. Among these 67 twins with informative X-inactivation profiles, 46 were from complete twin pairs (N = 23 pairs), whereas for the remaining 21 twins, only one of the twins in a pair had available informative X-inactivation data. In addition, 10 individuals from the 1895 centenarian study with X-inactivation data at age 100 participated in the yearly follow-up, and X-inactivation data were obtained at the latest participation year, i.e. 2–7 years later (Table 1).

**Table 1:** Degree of skewness (DS) of X-inactivation in two longitudinal studies; the septuagenarian twin cohort study (LSADT) and the centenarian study (1895 birth cohort).

	Longitudinal studies			
	LSADT		1895 Birth Cohort	
	Baseline	Follow-up	Baseline	Follow-up
Birth year	1917–1923		1895	
Interview year	1997	2007	1996–1997	1999–2004
Number of individuals	67	67	10	10
Mean Age, years	75	85	101	103–108
Age range, years	73–84	83–94	101	103–108
Skewed DS, 80 + (%)	25	36	60	60
Extremely skewed DS, 95+ (%)	7	10	20	20
Mean DS (SD)	71 (14)	72 (15)	82 (10)	80 (11)

When the septuagenarian and octogenarian women were first measured at baseline, 25% (95% CI 16–37%) of the women had skewed X-inactivation (DS 80+). After 10 years of follow-up 9 women changed status on skewness, and of these, 8 ascended to skewed X-inactivation with age (*P* = 0.04). This supports the sub-clonal expansion hypothesis of clonal expansion toward more skewness with age, but only in 8 of the 50 women (16%, 95% CI 7–29%) not already skewed at baseline. At extremely old ages, 6 of 10 centenarians (60%) had skewed X-inactivation (DS: 80+), but no differences were observed after 2–7 years of follow-up (see Table 1). This confirms that skewed X-inactivation increases with age, though it may not change longitudinally at extreme ages.

**Figure 2:**
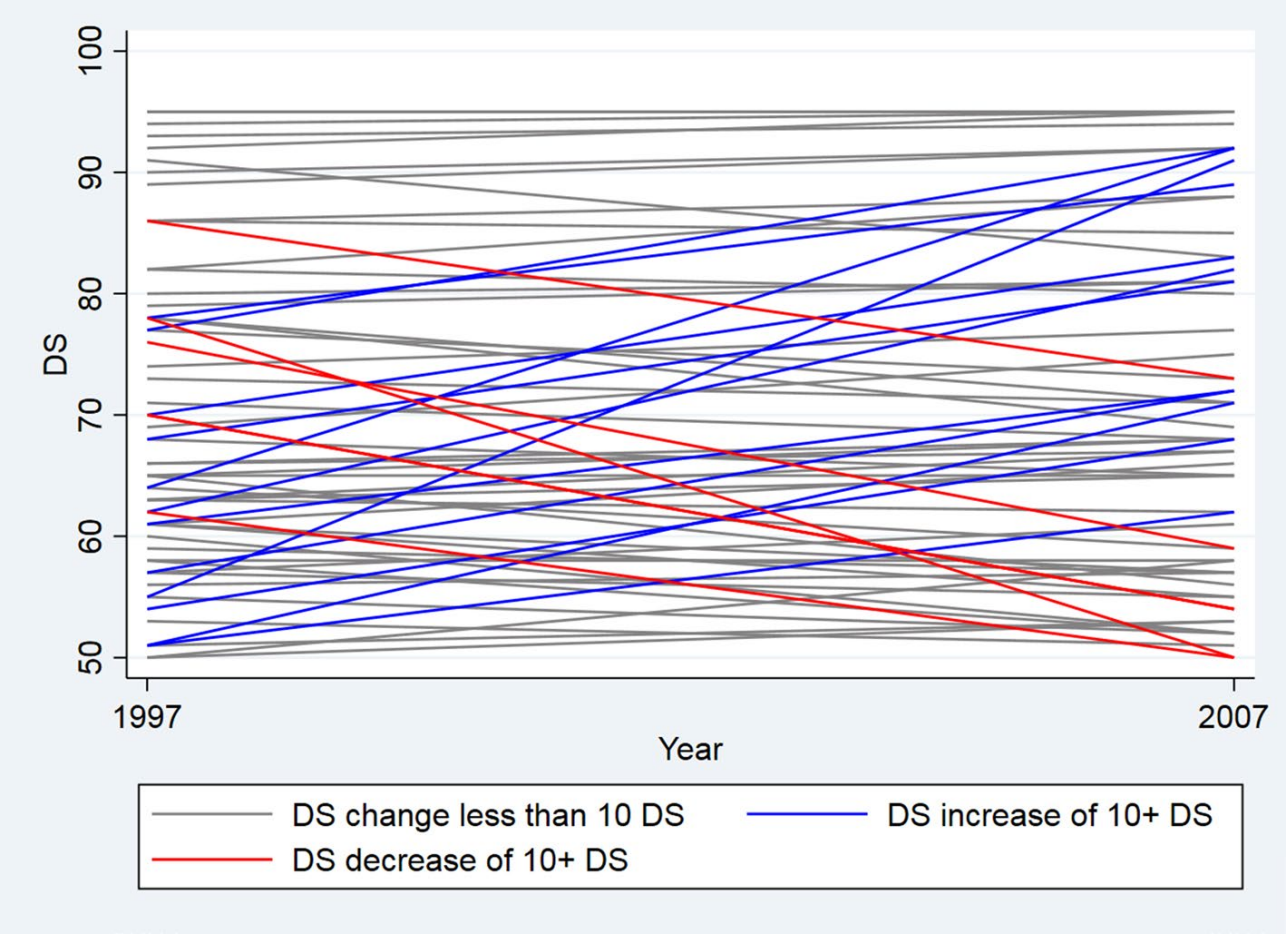
Longitudinal change in degree of skewed X-chromosome inactivation (DS) plotted at baseline in 1997 and follow-up in 2007 twin women (N = 67) from the LSADT cohort.

**Figure 3:**
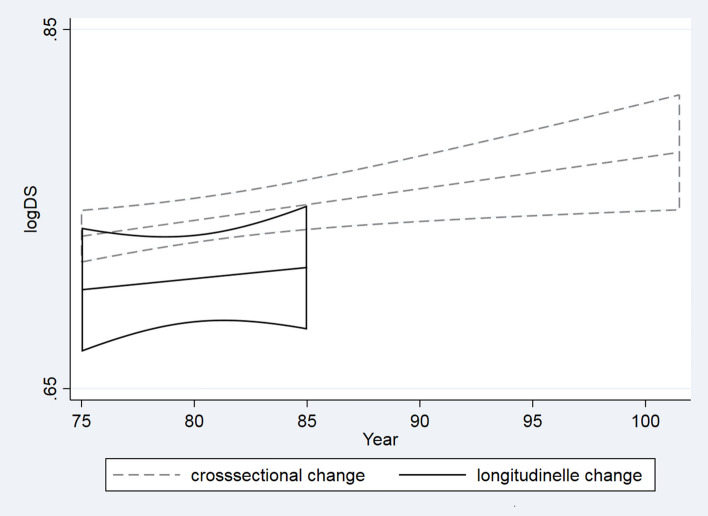
Cross-sectionally and longitudinally estimated changes in degree of skewed X-chromosome inactivation (DS) with age. Ten-year longitudinal changes of DS at baseline (1997) and follow-up (2007) in LSADT. Cross-sectional age changes were estimated using the LSADT (1997) and the 1905 birth cohort at 93 years of age and the 1895 centenarians.

Additional analyses supported the sub-clonal expansion hypothesis, as an increase in DS of more than 10 is more common (18%, 95% CI 0.10–0.29) than a decrease in DS of more than 10 (9%, 95% CI 0.03–0.18) (Fig. 2). However, there was no overall difference in DS between baseline and follow-up when adjustment for twin relation was made for septuagenarian and octogenarian twins (logitDS: 0.10, 95% CI (− 0.03 to 0.23)) or centenarian women (logitDS: − 0.14, 95% CI − 0.49 to 0.21). The non-significant yearly longitudinal mean DS increase (logitDS: 0.012, 95% CI (− 0.036 to 0.061)), here unadjusted, was 30% lower, based on the slope, compared to the cross-sectional mean increase with age (logitDS: 0.017, 95% CI (0.004–0.031)) (see Fig. 3), showing that the longitudinal increase with age is moderately lower than the cross-sectional increase with age.

**Figure 4:**
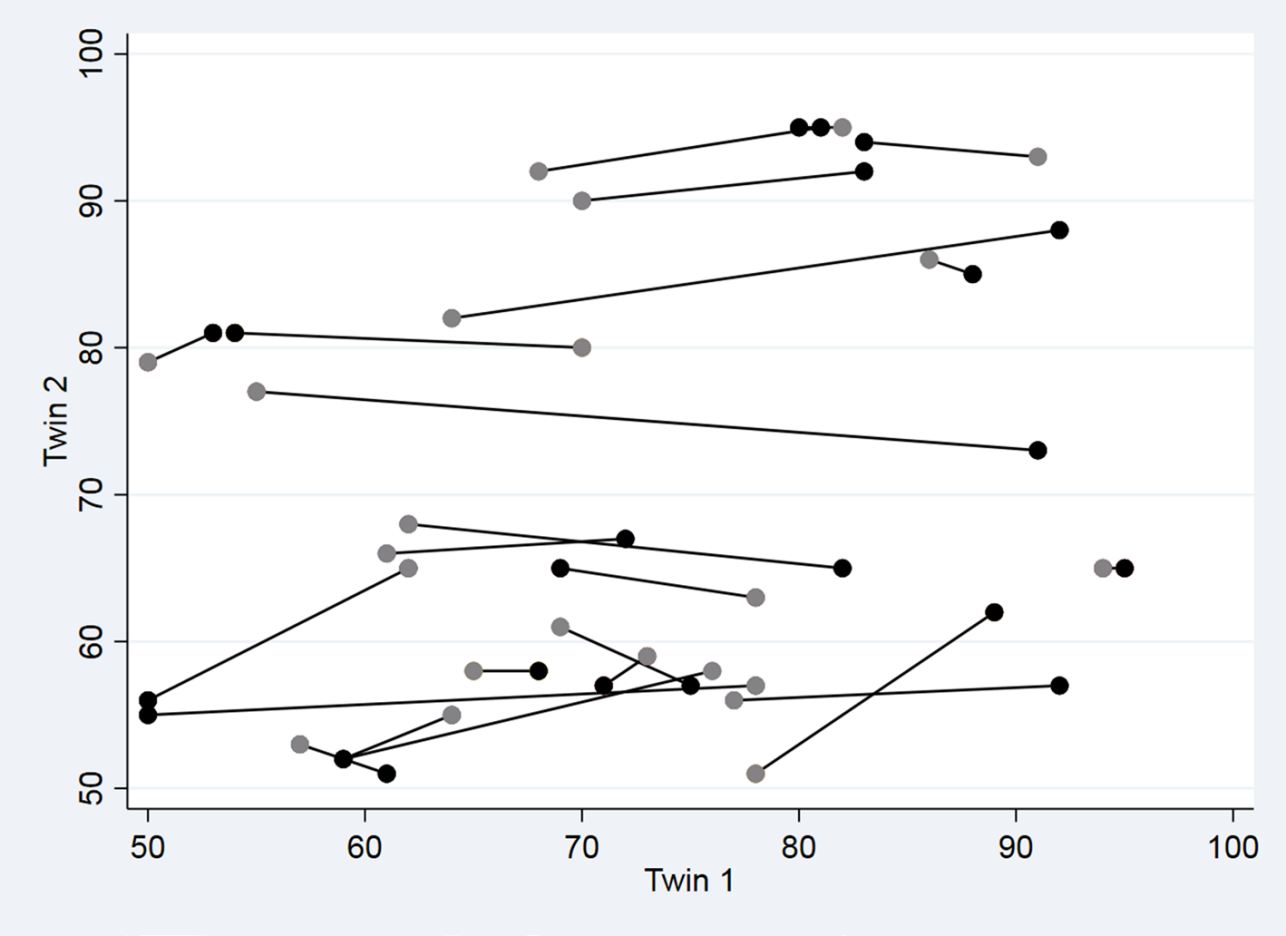
Co-twin correlations in degree of skewed X-chromosome inactivation (DS) within 23 twin pairs. Twin correlation of DS at baseline in 1997 (grey dots) and at follow-up in 2007 (black dots) with connected lines for each twin pair. Twin 1 is the twin with the largest numeric change in DS.

Among complete twin pairs, displayed in Fig. 4, recurrent skewed X-inactivation (DS 80+) in twin pairs at 10 years of follow-up occurred in 3 of 5 twin pairs (60%, 95% CI (15–95%)). In contrast, only 5 in 30 women (17%, 95% CI (6–35%)) ascended to skewed X-inactivation (DS 80+) in twin pairs without any skewed X-inactivation at baseline. The higher occurrence of skewed X-inactivation for co-twins of women with skewed X-inactivation was borderline significant when compared to women who had a co-twin with normal mosaics at baseline (*P* = 0.07). Of note, increasing or decreasing changes most often occur in only one of the twins of a pair, i.e. horizontal changes as opposed to diagonal changes, which represent simultaneous changes of DS with age, but which rarely occur in both twins of a pair in the same decade (Fig. 4). These data further support the hypothesis that changes in skewed X-inactivation in women occur individually, rather than as an overall longitudinal age-related fluctuation of skewed X-inactivation (e.g. pairwise) after 70 years of age.

### Cohort differences in skewed X-inactivation

Cohort differences in skewed X-inactivation were investigated in septuagenarians (73–79 years of age) born from year 1917 to 1923 and a younger cohort of septuagenarians, born from year 1931 to 1937. Furthermore, skewed X-inactivation was investigated at extremely old ages by comparing centenarians born in the years 1895, 1905 and 1915.

Here we found that among septuagenarian women from the oldest birth cohorts (1917–1923) 35% (95% CI (29–41%)) had skewed X-inactivation (DS 80+), whereas only 25% (95% CI (15–38%)) of women from the younger birth cohorts (1931–1937) had skewed X-inactivation (DS 80+). Similarly, more women had extremely skewed X-inactivation (DS 95 +) in the oldest birth cohorts compared to the younger cohort (Table 2). Also centenarians from the oldest cohort (the 1895 birth cohort) had higher DS than centenarians born in the younger cohorts. A total of 61% (95% CI (42–77%)) of women from the 1895 birth cohort, 43% (95% CI (32–55%)) from the 1905 birth cohort, and 45% (95% CI (34–55%)) from the 1915 birth cohort had skewed X-inactivation (DS 80+).

**Table 2:** Across-birth-cohort variation in degree of skewness (DS) of X-inactivation in age-matched septuagenarian twin cohorts (LSADT and MADT) and singleton centenarian cohorts (1895, 1905 and 1915 birth cohorts).

	Age-matched septuagenarian twins		Centenarian singletons		
	LSADT	MADT	1895 Birth Cohort	1905 Birth Cohort	1915 Birth Cohort
	Baseline	Baseline	Baseline	Baseline	Baseline
Birth year	1917–1923	1931–1937	1895	1905	1915
Blood draw year	1997	2008–2011	1996–1997	2005	2015
Number of individuals	234	59	33	81	98
Mean age, years, (range)	76 (73–79)	75 (73–79)	101	100	100
Skewed DS 80+ (%)	35	25	61	43	45
Extremely skewed DS, 95 + (%)	8	2	19	12	10
Mean DS (SD)	73 (14)	69 (13)	81 (11)	75 (14)	76 (14)

Additional analyses support across-cohort variation of skewed X-inactivation as septuagenarian women of the oldest birth cohorts (1917–1923) had higher DS than women from the younger birth cohort (1931–1937) (logit DS: 0.29 95% CI (0.04–0.54)), adjusted for twin correlation and technical variation. Also centenarians from the oldest cohort (the 1895 birth cohort) had higher DS than centenarians born in the younger cohorts combined (1905 birth cohort and 1915 birth cohort) (logit DS: 0.34 95% CI 0.01–0.67).

## Discussion

Skewed X-inactivation has in previous literature been shown to increases with age, but while the literature has mainly been based on cross-sectional studies, and while none of the rare longitudinal studies have reported longitudinal age-related changes (period changes), it is likely that the cross-sectionally observed age-related patterns could, in fact, be confounded by birth cohort differences. Here we demonstrate, in a longitudinal study, that while a fraction of 70 + year-old women (16%, 95% CI (7–29%)) changed from non-skewed to skewed during the 10 years of follow-up (period changes), the remaining sample had fixed DS during follow-up among septuagenarian, octogenarian and centenarian women. Moreover, for the first time, we found evidence of cohort effects in septuagenarians and centenarians born a decade or more apart, whereas more recent birth cohorts had lower DS. This indicates that not only cohort changes but also period changes (i.e. for a fraction of women) are driving the overall age-related increase in skewed X-inactivation seen in cross-sectional studies.

In the present work, we found no significant fluctuation in skewed X-inactivation during a follow-up period ranging from 2 to 10 years for the majority of women, but a fraction of them ascended to skewed X-inactivation after the age of 70. This is in line with a previous large study showing that only few samples had skewed X-inactivation in the blood at follow-up but not at intake, namely samples donated by women more than 60 years of age at follow-up^15^. In another small study, no fluctuation in skewed X-inactivation was found in blood samples from 20 to 75-year-old women^19^.

To our knowledge, the cohort effects on skewed X-inactivation observed in the present work are a novel observation that suggests that age differences in DS in cross-sectional studies are partly reflecting differences between birth cohorts. Other epigenetic studies have also shown age differences in cross-sectional studies; e.g., we have previously shown that methylation status changes with age on both sex chromosomes^26,27^, and that DNA-methylation clocks, e.g. the Horvath or Hannum clocks, show remarkably high correlation with chronological age^28–30^. How DNA-methylation and other epigenetic markers are related to skewed X-inactivation needs to be explored further, and, in particular, how the amount of somatic chromosomal loss and mutation load that increase with age may affect skewed X-inactivation; e.g., loss of sex chromosomes Y or X^1,2^, and clonal hematopoiesis^3^. A potential contribution to longitudinal changes in skewed X-inactivation in women might be that such age-related changes in cell composition and DNA structure affect skewed X-inactivation since women have the advantage of a back-up X-chromosome that may buffer such DNA alterations and thus potentially avoid harming hematopoietic stem cells.

Our finding that women aged 70 + from older birth cohorts had higher DS than women from more recent birth cohorts corresponds well with a mortality selection effect based on DS status^21^. If older birth cohorts have experienced a higher age-specific mortality than more recent birth cohorts based on their DS, then we will expect to see a higher fraction with the beneficial phenotype (i.e. high DS) in the older cohorts. For more recent birth cohorts, the probability of reaching higher ages is higher—maybe irrespective of DS or at least to lesser extents because of DS—than is the case for older birth cohorts. A reason for the cohort differences could be improved living conditions and better medical treatment in more recent birth cohorts^22,31^, potentially decreasing the effects of skewed X-inactivation in leukocytes. Alternatively, skewed X-inactivation may represent a marker of biological aging with more recent birth cohorts being biologically younger than older birth cohorts of the same chronological age, which is reflected in a lower degree of skewed X-inactivation in recent birth cohorts. As skewed X-inactivation is related to the immune system and only changes with age in blood cells^18^, it is reasonable to assume that immune biology is a major player in the biological aging theory suggested above.

The main advantage of this study is that an epigenetic marker in the form of skewed X-inactivation was studied in a unique sample of middle-aged, elderly and centenarian women from cohorts assessed at different calendar times. This allowed age-matched comparisons of septuagenarians and centenarians across birth cohorts, which has not been seen in previous studies. Also, studies using repeated measures over time are rare, especially at the extreme ages where mortality is very high. The longitudinal twin design also allowed us to study DS in age-matched individuals, and we found little correlation in DS- changes in related individuals over a decade of follow-up. Although family influence is likely caused by inherited genetics or somatic genetic variation, family-specific factors such as shared environment may also influence skewed X-inactivation. Future twin or family studies are therefore warranted to confirm these observations. A disadvantage of the study is that the longitudinal studies are selected for women in better than average health compared to women of the same age, and thus, women in poorer health, who are not included in the study, might modify the longitudinal change in DS.

In summary, skewed X-inactivation in peripheral blood increases with age in cross-sectional studies. Using a longitudinal and across-birth cohort study we show that not only age, but also birth cohort contributed to the age-gradient in degree of skewness. The reasons for the cohort differences could be improved living conditions and better medical treatment leading to improved survival in recent birth cohorts.

## Methods

### Surveys

The study population includes subjects from five population-based nationwide surveys conducted at the University of Southern Denmark: The Longitudinal Study of Aging Danish Twins (LSADT), The Middle-Aged Danish Twins (MADT) and The Danish 1895, 1905 and 1915 birth cohort studies. The cohorts are depicted in a lexis diagram (Fig. 1).

LSADT is a longitudinal study of Danish twins aged 70 years and older^32,33^. The study was initiated in 1995, and the survey was repeated in 1997, 1999, 2001, 2003, 2005 and 2007. In 1997, mainly twin pairs of the same sex were included in the study, and, moreover, both twins had to be alive and born before January 1924. Participants were thus between 73 and 95 years of age. Full blood samples were drawn in 1997 from 689 members of intact twin pairs and again in 2007 from the surviving 120 individuals from intact twin pairs. A total of 361 participants had X-chromosome inactivation patterns determined in the Oslo laboratory as previously described^14^.

The Middle-Aged Danish Twins were 46–67 years of age when the study was initiated in 1998^34^. Monozygotic, dizygotic and opposite sex twins born in each year between 1931 and 1952 were included in the cohort. The participants were re-visited from 2008 to 2011, and blood was donated during this re-assessment 10–14 years later^35^. Full blood samples from 500 women were included in the study and 59 were selected for the age matched cohort comparison.

The 1895 cohort is a longitudinal survey of an entire birth cohort. The survey was initiated in 1995–1996 and included consecutively all Danes turning 100 years (baseline). Follow-up was carried out at age 101.5, 103, 104, 105, 106, 107 and 108, which became the highest attained age of last two members of the birth cohort, whereafter the cohort was extinct. Samples from baseline (1996–1997) and the latest year of follow-up participation were included in the study^36^. The 1895 cohort comprised 276 centenarians (1995–1996) and of these 207 (75% women) participated in an interview in their own home or care home^37^. At age 101.5 years (1996–1997) 71 were interviewed and 62 donated blood (women; N = 45 73%), at age 103 (1998–1999) 24 were interviewed and 20 donated blood (women; N = 14 70%), at age 104 (1999–2000) 15 were interviewed and 16 donated blood (women; N = 11 73%), at age 105 (2000–2001) 9 were interviewed and 10 donated blood (women; N = 6 67%). At age 106 (2001–2002) 7 were interview and 6 donated blood (women; N = 5 71%), at age 107 (2002–2003) 5 were interviewed and 3 donated blood (women; N = 3 100%), and at age 108 (2003–2004) 1 woman donated blood. X-chromosome inactivation patterns were determined in the Oslo laboratory for 21 baseline (1996–1997) samples.

The Danish 1905 birth cohort study is a prospective investigation of an entire birth cohort (Nybo et al. 2003). The survey was initiated in 1998, when the participants were 92–93 years old and comprises follow-up studies in 2000, 2003 and 2005. In 2005, survivors (age 99–100 years, named centenarians) were invited to participate and 126 donated blood. Women participating in 2005 were included in this study^36^. The 1905 birth cohort centenarians comprised 439 eligible individuals of whom 256 (84% women) participated in an interview. Baseline DS (92–93 years old; n = 118) was used in cross-sectional age-related estimates, but too few samples from the same 1905 participants were measured both at baseline (1998) and follow-up (2005) to conduct longitudinal analyses on this population. Baseline (1998) X-chromosome inactivation patterns were determined in the Oslo laboratory.

The Danish 1915 West birth cohort study included all Danes born in 1915, alive on their 100th birthday and living in the Western part of Denmark. Centenarians were assessed in their own home or in care homes. This comprised 303 centenarians and of these 238 participated in an interview. Blood samples were collected at the time of the in-person interviews and 131 full blood samples could be collected. In addition, 4 centenarians from a pilot study performed in 2014, who were born in 1914, were included in the study. Samples from all women were included in the study^36^.

Written informed consent was obtained from all participants, and all studies were approved by the Regional Committees on Health Research Ethics for Southern Denmark under the following approval numbers: MADT (S-VF-19980072), LSADT (S-VF-20040241), the Danish 1895 and 1905 birth cohorts (VF-20040240) and the Danish 1915 birth cohort (S-20140099 and S-20100011). All methods used were in accordance with relevant guidelines and regulations.

### DNA extraction

DNA was extracted from total blood cells in peripheral full blood samples, which were packed by centrifugation at 1000*g* for 15 min followed by withdrawal of the plasma phase. The DNA was extracted by a standard manual salting out protocol including a protease digestion step^38^, or extracted using a manual salting out purification protocol (Qiagen).

### Analysis of X-chromosome inactivation

The X-chromosome inactivation pattern was determined by the HUMARA method^12^. In order to obtain a robust method, we used 50 ng DNA in the DNA digestion using the 10 U/µl HpaII or no enzyme in a 10 µl volume. DNA of 1 µl was added into a 25 µl reaction volume and PCR amplification was initiated by a denaturation at 94 °C followed by 36 cycles of 94 °C for 30 s, 60 °C for 30 s. and 72 °C for 60 s, followed by a 10 min. extension at 72 °C. Samples collected at baseline and follow-up (LSADT in 1997 and 2007, 1895 birth cohort in 1996–1997 and 1998–2004) for each sample were assayed and analyzed on the same plate and conducted in the Odense laboratory. Cross-sectional data were measured in women from MADT, 1895 centenarians, 1905 centenarians, and 1915 centenarians in the Odense laboratory whereas LSADT, 1905 (baseline) and a small sample (n = 21) of 1895 centenarians were measured in the Oslo laboratory. The DS was defined as a number between 50 and > 95, where 50 indicates a random X-inactivation pattern and > 95 an extremely skewed pattern, where most or all cells had either the maternal or the paternal X as the active X-chromosome. Differences in DS measures at different time points in two different laboratories (Odense, Denmark and Oslo, Norway) are displayed in supplementary Table S1, but only non-significant and marginal differences were seen. The total numbers of obtained informative DS measures are depicted in Fig. 1. For centenarians, all women who donated blood were included in the analyses, and the number added up to a total of 33, 81 and 98 women from the birth cohorts 1895, 1905 and 1915, respectively. Twin participants were selected randomly from blood donors as described above. Longitudinal DS measures were obtained from all women who donated blood at two assessments, and this added up to 67 twins (LSADT) and 10 centenarians (Table 1).

### Statistical analyses

Mean DS values and the standard deviation were estimated for each cohort, and also the common thresholds of skewed X-inactivation with a DS of 80 and above and extremely skewed X-inactivation with a DS of 95 and above were calculated for each cohort.

For testing associations, DS was, prior to analyses, logit transformed by the equation logit(DS/(1 − DS)) to meet the assumptions of a statistical inference in the mixed random effect model.

Associations between age and DS were assessed using a mixed random effect model assigning twin-pairing as a random variable. For the longitudinal analysis of DS after 10 years of follow-up, the logit transformed DS at baseline and DS at follow-up was coded 0 and 1. Estimations were carried out by a mixed random effect model with random effects of twin pairs and individual id. In the longitudinal analysis of DS for the 1895 centenarians, a mixed effect model with a random effect for individual id was used, DS at baseline and follow-up was coded 0 and 1 and introduced as random effect in the model. For each cohort, binomial proportion and 95% confidence intervals were estimated using the standard exact model. For comparing the within-twin pair longitudinal differences in DS (categorical data), Fisher’s exact test or an exact test (McNemar's) for categorical data (skewed X-inactivation DS 80+) was used. Technical duplicates in two independent labs showed no significant difference in the two sets of samples (LSADT and 1895), but both logit(DS) estimations were lower in the samples analyzed in the Odense laboratory. Technical variation did not affect the centenarian results that were all made in the Odense laboratory. The cross-sectional septuagenarian LSADT samples were assayed in the Oslo laboratory and the MADT samples in the Odense laboratory. The cohort effect between twins (MADT and LSADT) was estimated using a linear mixed model, with two level random effects of twin pairing and cohort technical variation. The cohort effects analysis between centenarian cohorts was done using a linear mixed model, without random effects. Statistical analyses were done using Stata 16.0 (Stockholm, Sweden).

## Supplementary Information

Supplementary Table S1.

## Data Availability

Data access: according to Danish legislation, transfer and sharing of individual-level data requires prior approval from the Danish Data Protection Agency and requires that data sharing requests be dealt with on a case-by-case basis. For this reason the data cannot be deposited in a public database. However, we welcome any enquiries regarding collaboration and individual requests for data sharing.
