# Supplementary material for: Skewness of X-chromosome inactivation increases with age and varies across birth cohorts in elderly Danish women
Source: Sci Rep. 2021 Feb 22;11:4326. doi: 10.1038/s41598-021-83702-2 (PMC7900237; doi:10.1038/s41598-021-83702-2)
Supplement: Supplementary file 1 — Supplementary Table S1. [file 41598_2021_83702_MOESM1_ESM.docx]

# Skewness of X-chromosome inactivation increases with age and varies across birth cohorts in elderly Danish women

by

Jonas Mengel-From, Rune Lindahl-Jacobsen, Marianne Nygaard, Mette Soerensen M, Karen Helene Ørstavik, Jens Michael Hertz, Karen Andersen-Ranberg, Qihua Tan and Kaare Christensen

Supplementary Table 1. Technical variation in degree of skewness (DS) was analysed between two laboratories (1) lab 1: Oslo, Norway and (2) lab 2: Odense, Denmark. Identical samples measured in the two laboratories were collected from the LSADT study and the 1895 centenarian study.

| Cohort | X-inactivation  status | No. samples | Lab 1 measures | Lab 2 measures | Difference in logDS* | P-value |
| --- | --- | --- | --- | --- | --- | --- |
| LSADT |  |  |  |  |  |  |
|  | mean DS (SD) | 67 | 72 (13.7) | 71 (13.7) | -.11 | 0.07 |
|  | DS 80+ (%) |  | 20 (30) | 17 (25) | -3 (5) | - |
|  | DS 95+ (%) |  | 6 (9) | 5 (7) | 1 (2) | - |
| 1895 centenarian cohort |  |  |  |  |  |  |
|  | mean DS (SD) | 21 | 82 (12.5) | 80 (11.5) | -.13 | 0.12 |
|  | DS 80+ (%) |  | 13 (62) | 10 (48) | -3 (13) | - |
|  | DS 95+ (%) |  | 5 (24) | 5 (23) | 0 | - |

* The difference in DS was calculated as the sum of the difference within each individual

SD: Standard Deviation
